# Distribution and Characteristics of Hypouricemia within the Japanese General Population: A Cross-Sectional Study

**DOI:** 10.3390/medicina55030061

**Published:** 2019-03-04

**Authors:** Shin Kawasoe, Kazuki Ide, Tomoko Usui, Takuro Kubozono, Shiro Yoshifuku, Hironori Miyahara, Shigeho Maenohara, Mitsuru Ohishi, Koji Kawakami

**Affiliations:** 1 Department of Pharmacoepidemiology, Graduate School of Medicine and Public Health, Kyoto University, Kyoto 606-8501, Japan; kawazoe.shin.85n@st.kyoto-u.ac.jp (S.K.); ide.kazuki.2r@kyoto-u.ac.jp (K.I.); usui.tmk@gmail.com (T.U.); 2 Center for the Promotion of Interdisciplinary Education and Research, Kyoto University, Kyoto 606-8501, Japan; 3 Department of Cardiovascular Medicine and Hypertension, Graduate School of Medical and Dental Sciences, Kagoshima University, Kagoshima 890-0075, Japan; kubozono@cepp.ne.jp (T.K.); ohishi@m2.kufm.kagoshima-u.ac.jp (M.O.); 4 Kagoshima Kouseiren Medical Health Care Center, Kagoshima 890-0062, Japan; hamuterupp@hotmail.com (S.Y.); hir-miya@dream.jp (H.M.); kou.maenohara@ks-ja.or.jp (S.M.)

**Keywords:** epidemiology, renal insufficiency, renal hypouricemia, sex difference

## Abstract

*Background and objectives:* There is insufficient epidemiological knowledge of hypouricemia. In this study, we aimed to describe the distribution and characteristics of Japanese subjects with hypouricemia. *Materials and Methods:* Data from subjects who underwent routine health checkups from January 2001 to December 2015 were analyzed in this cross-sectional study. A total of 246,923 individuals, which included 111,117 men and 135,806 women, met the study criteria. The participants were divided into quartiles according to their serum uric acid (SUA) levels. We subdivided the subjects with hypouricemia, which was defined as SUA level ≤ 2.0 mg/dL, into two groups and compared their characteristics, including their cardiovascular risks. *Results:* The hypouricemia rates were 0.46% overall, 0.21% for the men and 0.66% for the women (*P* < 0.001). The number of the subjects with hypouricemia showed two distributions at SUA levels of 0.4–1.1 mg/dL (lower hypouricemia group), which included a peak at 0.7–0.8 mg/dL, and at SUA levels of 1.4–2.0 mg/dL (higher hypouricemia group). The men in the higher hypouricemia group had lower body mass indexes (BMI) and triglyceride (TG) levels and had higher fasting blood glucose levels than those in the lower hypouricemia group. The women in the higher hypouricemia group were younger; had lower BMI, total protein, TG, total cholesterol and low-density lipoprotein cholesterol levels; and had higher estimated glomerular filtration rates levels compared to those in the lower hypouricemia group. *Conclusions:* The characteristics of the individuals in the lower and higher hypouricemia groups differed significantly, indicating different pathophysiologies within each group.

## 1. Introduction

Increases in uric acid clearance or decreases in uric acid production cause hypouricemia [1,2], which is defined as a serum uric acid (SUA) concentration ≤ 2.0 mg/dL [3,4]. Patients with hypouricemia do not usually display any symptoms or complications and hypouricemia is often detected incidentally during routine health checkups.

Renal hypouricemia (RHUC) is caused mainly by urate transporter gene mutations, including SLC22A12 (URAT1) [5] and SLC2A9 (GLUT9/URATv1) [6]. This is important to note due to the risks associated with exercise-induced acute kidney injury [4]. Recently, the Japanese Society of Gout and Nucleic Acid Metabolism produced the first guidelines on RHUC, which recommend definitive diagnoses through more detailed examinations that should include measurements of the urinary excretion of urate and ruling out other uric acid-lowering diseases if patients have SUA levels ≤ 2.0 mg/dL. High SUA levels have been associated with elevated risks for cardiovascular events and mortality [7,8]. In contrast, the findings of some studies have shown increased risks for cardiovascular events among subjects with low SUA levels, resulting in a “J-shaped” association between the SUA levels and the cardiovascular risks. The precise mechanisms that underlie these cardiovascular risks and the SUA values that are associated with the lowest cardiovascular risks remain unclear.

Our knowledge about hypouricemia remains inadequate because it is uncommon and thus, more detailed epidemiological descriptions are essential to facilitate investigations into the pathophysiology of hypouricemia. Based on the results from a previous study [9], we developed the following hypotheses: (1) there are two independent distributions of individuals with hypouricemia, with the lower and higher hypouricemia groups having different characteristics; (2) the characteristics of the lower hypouricemia group do not differ from those of subjects who do not have hypouricemia; and (3) the higher hypouricemia group has the lowest cardiovascular risk. Herein, to describe the distribution and characteristics of Japanese subjects with hypouricemia, we conducted a descriptive analysis using data from a large number of subjects who underwent routine health checkups.

## 2. Materials and Methods

### 2.1. Study Population

**Figure 1:**
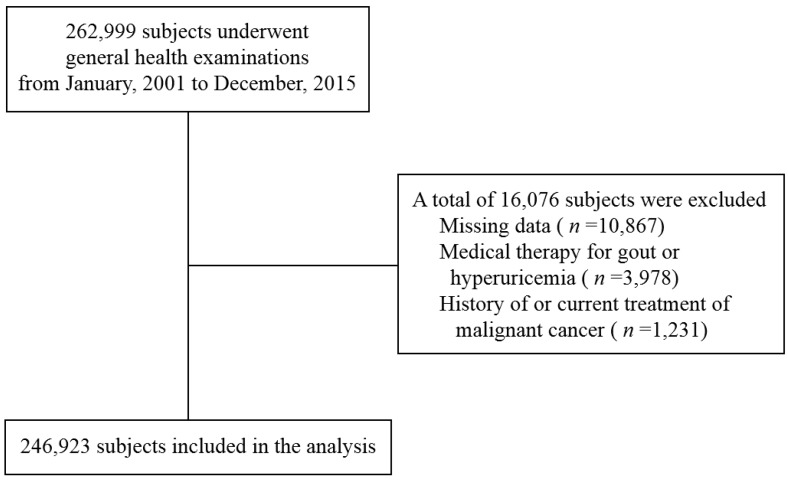
Flow diagram for selection of subjects. Among the 262,999 persons, 246,923 were included in the analysis.

The data from subjects who underwent routine health checkups from January 2001 to December 2015 at the JA Kagoshima Kouseiren Medical Health Care Center were analyzed in this cross-sectional study. Individuals who were receiving treatment for gout or hyperuricemia and those who had histories of or were undergoing treatment for malignancies were excluded. We also excluded subjects who did not have data that described their SUA levels or the other variables used in the analyses. Figure 1 shows the flow diagram for the subjects in this study. We analyzed 246,923 individuals, which included 111,117 men and 135,806 women. If the subjects had been examined more than once during the period studied, we only used the results from the first examination to avoid double counting.

This study conformed to the principles of the Declaration of Helsinki and it was approved by the institutional ethics committees of Kyoto University’s Graduate School of Medicine (No. R1443) and JA Kagoshima Kouseiren Hospital (No. 168).

### 2.2. Data Collection

Blood pressure was measured using a mercury sphygmomanometer after the subjects had sat quietly for 5 min. Blood samples were obtained from the subjects while they fasted. The levels of SUA and those of other biochemical parameters, including aspartate transaminase (AST), alanine aminotransferase (ALT), total bilirubin, total protein, creatinine, triglyceride (TG), low-density lipoprotein cholesterol (LDL-C), high-density lipoprotein cholesterol (HDL-C) and fasting blood glucose (FBG), were measured using standard laboratory procedures. Hypouricemia was defined as an SUA level of ≤ 2.0 mg/dL [3]. The estimated glomerular filtration rate (eGFR) was determined using the new Japanese coefficient for the modified isotope dilution mass spectrometry-traceable Modification of Diet in Renal Disease study equation:

eGFR = 194 × serum creatinine level^−1.094^ × age ^−0.287^ (1)

For women, the eGFR was multiplied by a correction factor of 0.739 [10].

A self-administered questionnaire was used to obtain information about an individual’s cigarette smoking status that was categorized as smoker or nonsmoker, which included never smoker or past smoker. Furthermore, we used this questionnaire to determine their alcohol drinking status, which categorized them as an infrequent drinker defined as drinking on ≤ 10 days/month or a frequent drinker defined as drinking on >10 days/month. Data regarding current medication for hypertension, diabetes mellitus and dyslipidemia were also obtained using the questionnaire.

### 2.3. Statistical Analysis

We divided the subjects into quartiles according to their SUA levels. Within the first quartile, the subjects with hypouricemia that was defined as an SUA level ≤ 2.0 mg/dL were subcategorized into two subgroups, which were namely the lower hypouricemia group and higher hypouricemia group, because two distinct prevalence distributions were evident. The cutoff point between the groups was the SUA value that corresponded to the lowest number of subjects between the two prevalence distributions.

As the SUA levels differ substantially between sexes, men and women were analyzed separately. The continuous variables are expressed as the means and the standard deviations, except for the AST, ALT and TG levels that are expressed as the medians [first quartiles, third quartiles]. The categorical variables are expressed as numbers and percentages. We showed the characteristics in each group and described those with and without hypouricemia.

We compared these groups using one-way analysis of variance for all continuous variables, except for the AST, ALT and TG levels for which we used the Kruskal–Wallis test and the chi-squared test for the categorical variables. After this, we compared the characteristics of the subjects in the lower and higher hypouricemia groups using Student’s unpaired t-test for all the continuous variables, except for the AST, ALT and TG levels for which we used the Wilcoxon signed-rank test and the chi-squared test for the categorical variables. Furthermore, we compared the subjects in the lower hypouricemia group or those in the higher hypouricemia group with the subjects who did not have hypouricemia to determine whether the lower hypouricemia group or higher hypouricemia group had particular characteristics.

The statistical analyses were performed using JMP Pro, version 11 (SAS Institute Inc., Cary, USA) for Windows. *P* values < 0.05 were considered to be statistically significant.

## 3. Results

### 3.1. Prevalence of Hypouricemia

The hypouricemia rates were 0.46% (*n* = 1135) overall, 0.21% (*n* = 235) for the men and 0.66% (*n* = 900) for the women (*P* < 0.001).

### 3.2. Two Hypouricemia Distributions

**Figure 2:**
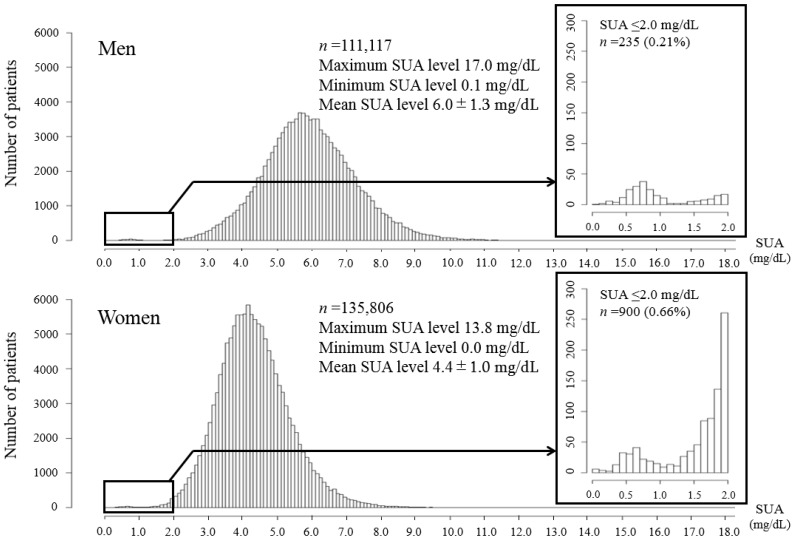
Distributions of the participants in relation to the serum uric acid levels according to sex.

Figure 2 presents the distribution of the subjects in relation to the SUA levels according to sex. Two distributions of prevalence occurred at SUA levels ≤ 2.0 mg/dL that were evident for both sexes. One group of individuals was distributed around SUA levels of 0.4–1.1 mg/dL, with a peak at 0.7–0.8 mg/dL. The other group of individuals corresponded to SUA levels ≥ 1.4 mg/dL and the numbers of individuals rose gradually to a peak at 2.0 mg/dL and continued smoothly to SUA levels ≥ 2.1 mg/dL.

In the region of the graphs where the SUA levels were ≤ 2.0 mg/dL, there are two visible distributions. One group of individuals was distributed around SUA levels of 0.4–1.1 mg/dL, with a peak at 0.7–0.8 mg/dL. The other group of individuals corresponded to SUA levels ≥ 1.4 mg/dL and the numbers of individuals rose gradually to a peak at 2.0 mg/dL and plateaued at SUA levels > 2.1 mg/dL. SUA = serum uric acid

Between the two distributions, the lowest number of individuals corresponded to an SUA level of 1.3 mg/dL. Using this value as the cutoff point, we divided the subjects with SUA ≤ 2.0 mg/dL into two groups, which were namely the lower hypouricemia group with SUA levels of 0.0–1.3 mg/dL and the higher hypouricemia group with SUA levels of 1.4–2.0 mg/dL. The lower hypouricemia group was comprised of 173 men (0.16%) and 221 women (0.16%) (*P* = 0.577) and the higher hypouricemia group was comprised of 62 men (0.06%) and 679 women (0.51%) (*P* < 0.001).

### 3.3. Characteristics of the Study Population Categorized According to the Serum Uric Acid Levels

Table 1 shows the clinical characteristics of the subjects categorized according to their SUA levels. As the SUA levels rose from 1.4–2.0 mg/dL to the fourth quartile, consistent positive trends were evident for both sexes regarding the body mass index (BMI) and TP, AST, ALT and TG levels while consistent negative trends were apparent regarding the eGFRs and HDL-C levels. As the SUA levels rose from 1.4–2.0 mg/dL to the fourth quartile, the mean age gradually declined among the men and it increased among the women. All analyzed variables showed different trends in SUA levels of 0.0–1.3 mg/dL.

The results of the comparison between the subjects with hypouricemia and without hypouricemia are shown in Table 2. In men, the subjects with hypouricemia were older and had lower levels of TC and higher levels of FBG and eGFR compared with those without hypouricemia. In women, the subjects with hypouricemia were younger and had lower levels of BMI, SBP, TP, ALT, TC, TG and LDL-C; lower proportion of medication for hypertension and dyslipidemia; higher levels of AST, HDL-C, FBG and eGFR; and higher proportion of medication for diabetes compared with those without hypouricemia.

### 3.4. Characteristics in the Lower and Higher Hypouricemia Groups

Compared with the women in the lower hypouricemia group, those in the higher hypouricemia group were significantly younger and their BMIs and TP, AST, ALT, TC, TG and LDL-C levels were lower, their eGFRs were higher and fewer drank alcohol frequently (Table 3; comparison I). The men in the higher hypouricemia group had significantly lower BMIs and lower TG levels than those in the lower hypouricemia group (Table 3; comparison I). The characteristics of the male and female subjects in the lower hypouricemia groups and those of the individuals without hypouricemia did not differ significantly (Table 3; comparison II). In men, compared with those without hypouricemia, the subjects in the higher hypouricemia group were older and had lower levels of BMI and TG; higher levels of FBG and eGFR; and higher proportion of medication for diabetes. In women, the subjects with higher hypouricemia were younger and had lower levels of BMI, SBP, TP, AST, ALT, TC, TG and LDL-C; lower proportion of frequent alcohol drinkers and medication for hypertension; higher levels of HDL-C, FBG and eGFR; and higher proportion of medication for diabetes compared with those without hypouricemia (Table 3; comparison III).

### 3.5. Possible Cardiovascular Risks Associated with the Bottom of the J-Shaped Curve

**Figure 3:**
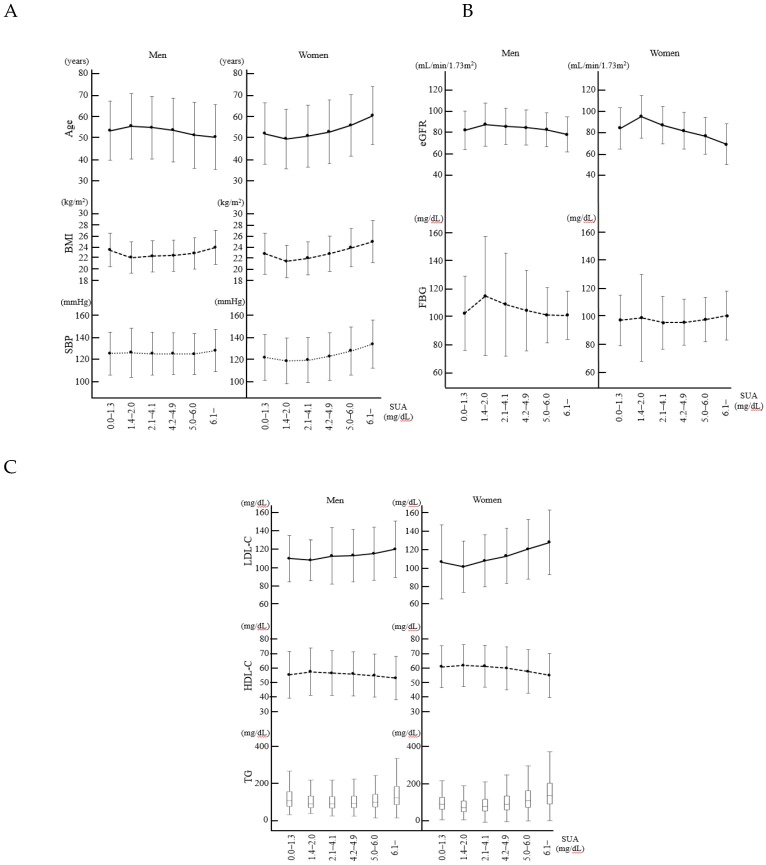
Characteristics of each serum uric acid category in the context of cardiovascular risks according to sex. The graphs in panels (**A**–**C**) show the variables associated with cardiovascular risk for each SUA category according to sex. Among the male participants, age, estimated glomerular filtration rate (eGFR), fasting blood glucose (FBG) and high-density lipoprotein cholesterol (HDL-C) levels were the highest while the body mass index (BMI) and the low-density lipoprotein cholesterol (LDL-C) and triglyceride (TG) levels were lowest in the SUA 1.4–2.0 mg/dL category. Among the female participants, the eGFR, FBG and HDL-C levels were the highest while age, BMI, systolic blood pressure and LDL-C and TG levels were the lowest in the 1.4–2.0 mg/dL category. The data presented are the means and standard deviations, except for the TG values that are presented as the median and the first and third quartiles. BMI = body mass index; eGFR = estimated glomerular filtration rate; FBG = fasting blood glucose; HDL-C = high-density lipoprotein cholesterol; LDL-C = low-density lipoprotein cholesterol; SUA = serum uric acid; SBP = systolic blood pressure; TG = triglyceride.

The characteristics of the individuals in each of the SUA categories according to sex are illustrated in Figure 3. Age, eGFR and FBG and HDL-C levels were at their highest while BMI and LDL-C levels were at their lowest in male subjects in the SUA 1.4–2.0 mg/dL category. The eGFR and HDL-C levels were at their highest while age, BMI and systolic blood pressure, LDL-C and TG levels were at their lowest in female subjects in the 1.4–2.0 mg/dL category.

## 4. Discussion

This is the first study to analyze over 1000 people with hypouricemia and to subdivide the study cohort for in-depth analyses of their characteristics. The prevalence of hypouricemia is low. To investigate the distribution and characteristics of patients with hypouricemia, we needed to obtain sufficient number of subjects with hypouricemia and conduct research on such populations. The study’s main findings showed that (1) the hypouricemia rates were 0.46% overall, 0.21% for men and 0.67% for women (*P* < 0.001); () the prevalence of hypouricemia peaked at SUA levels of 0.0–1.3 mg/dL and 1.4–2.0 mg/dL and the characteristics of the individuals in these subgroups differed; (3) the characteristics of the subjects whose SUA levels were 0.0–1.3 mg/dL and those without hypouricemia did not differ; and (4) subjects of both sexes whose SUA levels were 1.4–2.0 mg/dL had the lowest BMIs and LDL-C and TG levels and the highest HDL-C levels and eGFRs.

### 4.1. Prevalence of Hypouricemia

The prevalence of hypouricemia, which was defined as ≤ 2.0 mg/dL, was 0.51% among outpatients in Turkey [11] and 0.53% in Korea [12]. In Japan, Wakasugi et al. reported that 0.21% of men and 0.39% of women within the general population had hypouricemia [13]. Kuwabara et al. reported that the prevalence of hypouricemia in the general population showed regional differences in Japan and 0.068% of men and 0.310% of women in Tokyo had hypouricemia, while 0.318% of men and 1.237% of women in Yonago had hypouricemia [9]. The findings from these studies suggest that differences exist regarding the prevalence of hypouricemia according to race and region and that compared with men, the prevalence of hypouricemia is 2–4-fold higher in women. The prevalence of hypouricemia in this study differed from the rates reported previously. However, the ratio of men-to-women who had hypouricemia concurred with those reported previously.

A particularly noteworthy finding from this study was the peak in prevalence that occurred at SUA levels of 0.6–1.3 mg/dL in both sexes. The findings from a previous study also showed a peak at similar SUA levels [9]. We speculate that different pathophysiologies underlie hypouricemia at SUA levels of 0.6–1.3 mg/dL and at higher SUA levels. In this study, the subjects were assigned to the lower hypouricemia group (SUA level 0.0–1.3 mg/dL) or the higher hypouricemia group (SUA level 1.4–2.0 mg/dL). Similar proportions of men and women were present in the lower hypouricemia group. Sugihara et al. investigated SCL22A12 mutations in 26 hypouricemic patients and found that all patients with SUA levels < 1.3 mg/dL had SLC22A12 mutations and that the male-to-female ratio was almost equal [14]. Reports from other studies have not described preponderances of men among patients with RHUC [15] or among asymptomatic patients with SLC22A12 mutations [16,17]. The present study’s almost equal male-to-female ratio in the lower hypouricemia group suggests that this group included individuals with the SLC22A12 mutation. It seems unlikely that sex differences exist regarding hypouricemia because the SLC22A12 and SLC2A9 genes are on autosomal chromosomes. In contrast, the higher hypouricemia subgroup in this study had different percentages of men (0.06%) and women (0.51%) (*P* < 0.001). The previously described disparities between men and women regarding the prevalence of hypouricemia may relate to differences in the numbers of individuals with SUA levels of 1.4–2.0 mg/dL.

### 4.2. Difference of the Characteristics between the Two Groups of Hypouricemia

For the first time, we have provided a detailed description of the characteristics of subjects with SUA levels ≤ 2.0 mg/dL. We found a discontinuous peak in the distribution of the individuals at SUA levels of 0.6–1.3 mg/dL. In contrast, the proportion of subjects with SUA levels of 1.4–2.0 mg/dL increased successively as the SUA levels rose and they continued to increase to peaks at SUA levels of 6.0 mg/dL for men and 4.4 mg/dL for women.

By comparing the lower and higher hypouricemia groups with respect to some demographic and clinical characteristics, we verified that the characteristics of individuals in these groups differed. Many baseline characteristics differed between the women in the lower hypouricemia group and those in the higher hypouricemia group. Compared with those in the higher hypouricemia group, the subjects in the lower hypouricemia group were significantly older, their BMIs, systolic blood pressure and TG and LDL-C levels were higher and their eGFRs were lower. Interestingly, no differences were found between subjects in the lower hypouricemia group and those without hypouricemia, indicating that the characteristics of the subjects whose SUA levels were 0.0–1.3 mg/dL were similar to those of the individuals without hypouricemia. Hence, we speculate that the subjects with SUA levels of 0.0–1.3 mg/dL may have had RHUC, which is caused by urate transporter gene mutations and that they did not differ from the subjects in the higher quartiles, except for their extremely low SUA levels. In contrast, transient uric acid-lowering factors, for example, vegetarian diets [18,19], medication [20] and medical conditions, including diabetes and liver disease [21], may have been associated with the hypouricemia in subjects with SUA levels of 1.4–2.0 mg/dL.

The men in the lower and higher hypouricemia groups showed statistically significant differences in relation to a limited number of variables only, regardless of the differences in the numerical values; this may have been a consequence of the low level of statistical power that was caused by the low number of individuals in the higher hypouricemia group. If we compared the characteristics of subjects with 0.0–1.3 mg/dL with those with SUA 1.4–2.5 mg/dL, the subjects with SUA 0.0–1.3 mg/dL showed significantly higher BMI and TG and lower age, HDL-C, FBS levels and eGFR compared with those with SUA 1.4–2.5 mg/dL (Table S1). In contrast, no differences were observed between the men in the lower hypouricemia group and those without hypouricemia. The women showed similar results, which suggests that individuals whose SUA levels are 0.0–1.3 mg/dL may have RHUC and that other uric acid-lowering factors may be involved in those with SUA levels of 1.4–2.0 mg/dL.

### 4.3. SUA Values Associated with the Bottom of Cardiovascular Risks

Several investigators have reported that the association between SUA levels and risk of cardiovascular events or mortality follows a J-shaped curve. Verdecchia et al. reported that SUA levels < 4.5 mg/dL in men and < 3.2 mg/dL in women were associated with increased risks of cardiovascular diseases [22]. Mazza et al. showed that subjects with SUA levels < 4.9 mg/dL had increased risks of cardiovascular mortality [23]. Although uric acid is one of the most powerful antioxidants and scavengers of singlet oxygen and radicals [24], the precise mechanisms underlying the association between low SUA levels and the increase in cardiovascular risks remain unclear. Furthermore, the SUA value at the bottom of the J-shaped curve has not been elucidated.

Regarding the established cardiovascular risk factors, including the BMI, serum lipid profiles and renal function, the results from the current study suggest that SUA levels at the bottom of the J-shaped curve were between 1.4 mg/dL and 2.0 mg/dL. In contrast, the subjects in the lower hypouricemia group had different cardiovascular risk factor profiles that were similar to those of the subjects without hypouricemia. It is possible that the previously reported elevations of cardiovascular risks in groups of individuals with low SUA levels, which were namely < 3.0 mg/dL or < 4.0 mg/dL, were caused by the inclusion of individuals with SUA levels of 0.0–1.3 mg/dL in the analysis, which would have resulted in the risks appearing to be greater than those at slightly higher SUA levels, thereby producing a J-shaped curve. Even if the results from the previous studies had been adjusted for some confounding factors, others would not have been considered, such as inflammation [25], small dense LDL-C [26] and lipoprotein (a) [27]. Further studies are warranted to explore the association between low SUA levels and cardiovascular risks.

### 4.4. Study Limitations

Our study has several limitations. First, the subjects underwent health checkups at a single facility only. Second, this study was cross-sectional, SUA levels and other confounding factors were only measured once and we did not consider the variations associated with the measurement of each uric acid value. However, it is notable that the study involved a large number of individuals, accounting for approximately 20% of the adults in the Kagoshima Prefecture and we collected data from over 1000 individuals with hypouricemia. Third, we did not measure urinary excretion of uric acid. Therefore, we could not determine whether the hypouricemia was caused by a decrease in the production of uric acid or an increase in its urinary excretion. Fourth, since there was no consensus on how to decide the cutoff value in two different distributions, we adopted the uric acid value in this study, which contained the lowest proportion of subjects with hypouricemia, as the cutoff point of two distributions in hypouricemia. Finally, we did not include data that described medications, such as diuretics and renin-angiotensin system inhibitors, or pregnancy status and menopause in women that may affect serum UA levels.

## 5. Conclusions

We have described the distribution of hypouricemia within the Japanese general population in detail and analyzed the characteristics of hypouricemic individuals. The distribution of the individuals formed two peaks within the range of SUA levels that were defined as hypouricemia and the characteristics of the individuals in these two groups differed, which suggests differences regarding the pathophysiology of hypouricemia. Further studies are warranted to explore the precise pathophysiology of hypouricemia and the influence of hypouricemia on cardiovascular events.

## Figures and Tables

**Table 1:** Clinical characteristics of the male and female participants categorized according to their serum uric acid levels.

**Men**							
	**SUA**						
	**First Quartile**			**Second Quartile**	**Third Quartile**	**Fourth Quartile**	***P* Value**
	**Lower Hypouricemia Group**	**Higher Hypouricemia Group**					
	**0.0–1.3 mg/dL**	**1.4–2.0 mg/dL**	**2.1–4.1 mg/dL**	**4.2–4.9 mg/dL**	**5.0–6.0 mg/dL**	**≥6.1 mg/dL**	
	***n* = 173**	***n* = 62**	***n* = 8402**	***n* = 14,918**	***n* = 36,811**	***n* = 50,751**	
Age (years)	53.5 ± 13.7	55.6 ± 15.2	54.9 ± 14.5	53.7 ± 14.8	51.4 ± 15.3	50.5 ± 15.0	<0.001
BMI (kg/m^2^)	23.4 ± 3.1	22.0 ± 2.9	22.3 ± 2.9	22.3 ± 2.9	22.8 ± 2.9	23.9 ± 3.2	<0.001
SBP (mmHg)	124.9 ± 20.2	125.7 ± 23.1	124.7 ± 20.0	124.6 ± 19.6	124.5 ± 19.4	127.5 ± 19.8	<0.001
TP (g/dL)	7.5 ± 0.5	7.4 ± 0.4	7.4 ± 0.5	7.4 ± 0.5	7.5 ± 0.5	7.6 ± 0.5	<0.001
AST (IU/L)	25 {20,31}	23 {18,30.25}	23 {19,28}	23 {19,28}	23 {19,29}	25 {21,32}	<0.001
ALT (IU/L)	19 {14,30.5}	18 {13.75,24.25}	18 {13,24}	18 {13,24}	18 {14,26}	22 {15,32}	<0.001
T-bil (mg/dL)	0.83 ± 0.38	0.84 ± 0.45	0.82 ± 0.36	0.80 ± 0.35	0.81 ± 0.36	0.81 ± 0.36	0.065
eGFR (mL/min.1.73 m^2^)	81.9 ± 18.6	87.5 ± 20.6	85.5 ± 17.4	84.5 ± 16.7	82.4 ± 16.4	78.1 ± 17.0	<0.001
TC (mg/dL)	193.1 ± 35.4	193.6 ± 36.3	193.5 ± 34.8	193.7 ± 34.4	195.2 ± 34.6	202.1 ± 36.5	<0.001
TG (mg/dL)	104 {73.5,152.5}	88 {65.75,128}	88 {65.5,125}	89 {66,128}	95 {69,138}	119 {82,182}	<0.001
LDL-C (mg/dL)	109.3 ± 25.8	107.4 ± 22.9	112.2 ± 31.5	112.6 ± 29.2	114.8 ± 29.5	119.8 ± 31.4	<0.001
HDL-C (mg/dL)	55.5 ± 15.8	57.5 ± 15.9	56.6 ± 14.9	56.1 ± 14.8	55.0 ± 14.6	53.2 ± 14.6	<0.001
FBG (mg/dL)	102.4 ± 26.9	114.9 ± 43.4	108.7 ± 37.7	104.3 ± 29.5	101.0 ± 20.1	100.9 ± 17.5	<0.001
Current smoking, n (%)	77 (44.5)	25 (40.3)	3,649 (43.4)	6,835 (45.8)	16,926 (46.0)	22,416 (44.2)	<0.001
Frequent alcohol drinker, n (%)	97 (56.1)	32 (51.6)	4,396 (52.3)	7,944 (53.2)	20,480 (55.6)	31,870 (62.8)	<0.001
Medication for hypertension, n (%)	18 (10.4)	9 (14.5)	776 (9.2)	1,300 (8.7)	3,454 (9.4)	6,581 (13.0)	<0.001
Medication for diabetes, n (%)	4 (2.3)	5 (8.1)	368 (4.4)	526 (3.5)	779 (2.1)	664 (1.3)	<0.001
Medication for dyslipidemia, n (%)	5 (2.9)	0 (0.0)	107 (1.3)	150 (1.0)	408 (1.1)	797 (1.6)	<0.001
**Women**							
	**SUA**						
	**First quartile**			**Second Quartile**	**Third Quartile**	**Fourth Quartile**	***P* Value**
	**Lower Hypouricemia Group**	**Higher Hypouricemia Group**					
	**0.0–1.3 mg/dL**	**1.4–2.0 mg/dL**	**2.1–4.1 mg/dL**	**4.2–4.9 mg/dL**	**5.0–6.0 mg/dL**	**≥6.1 mg/dL**	
	***n* = 221**	***n* = 679**	***n* = 58,697**	***n* = 40,966**	***n* = 26,941**	***n* = 8,302**	
Age (years)	52.1 ± 14.2	49.6 ± 13.7	51.0 ± 14.2	52.9 ± 14.7	55.9 ± 14.2	60.3 ± 13.3	<0.001
BMI (kg/m^2^)	22.8 ± 3.7	21.4 ± 2.9	22.0 ± 3.0	22.8 ± 3.2	23.9 ± 3.5	25.0 ± 3.8	<0.001
SBP (mmHg)	121.7 ± 20.6	118.6 ± 20.7	119.4 ± 20.3	122.7 ± 21.3	127.7 ± 21.8	133.8 ± 21.9	<0.001
TP (g/dL)	7.6 ± 0.5	7.5 ± 0.5	7.6 ± 0.5	7.6 ± 0.5	7.7 ± 0.5	7.8 ± 0.5	<0.001
AST (IU/L)	21 {17,25}	19 {16,23}	20 {17,24}	21 {18,25}	22 {19,27}	24 {20,29}	<0.001
ALT (IU/L)	15 {11,20}	12 {9,17}	13 {10,17}	14 {11,19}	16 {12,22}	18 {13,25}	<0.001
T-bil (mg/dL)	0.75 ± 0.28	0.74 ± 0.33	0.74 ± 0.30	0.74 ± 0.30	0.73 ± 0.29	0.72 ± 0.29	<0.001
eGFR (mL/min/1.73 m^2^)	83.4 ± 19.4	94.3 ± 19.9	86.3 ± 17.7	81.1 ± 17.3	76.3 ± 17.2	68.3 ± 19.3	<0.001
TC (mg/dL)	204.3 ± 35.2	195.2 ± 34.7	200.6 ± 35.6	206.8 ± 36.4	213.5 ± 37.2	219.8 ± 38.5	<0.001
TG (mg/dL)	84 {62.5,111}	70 {54,96}	75 {56,103}	84 {61,117}	98 {71,138}	118 {85,169}	<0.001
LDL-C (mg/dL)	105.8 ± 41.4	100.5 ± 28.8	107.2 ± 29.3	112.6 ± 30.9	120.4 ± 33.5	128.1 ± 36.2	<0.001
HDL-C (mg/dL)	61.1 ± 14.2	62.0 ± 14.2	61.5 ± 14.2	60.1 ± 14.5	58.0 ± 14.6	55.3 ± 14.9	<0.001
FBG (mg/dL)	97.7 ± 18.6	99.5 ± 31.9	96.0 ± 19.5	96.3 ± 16.7	98.1 ± 16.2	101.0 ± 17.9	<0.001
Current smoking, n (%)	12 (5.4)	34 (5.0)	2,776 (4.7)	2,123 (5.2)	1,426 (5.3)	468 (5.6)	<0.001
Frequent alcohol drinker, n (%)	24 (10.9)	42 (6.2)	4,362 (7.4)	3,641 (8.9)	2,676 (9.9)	909 (11.0)	<0.001
Medication for hypertension, n (%)	30 (13.6)	66 (9.7)	4,661 (7.9)	5,003 (12.2)	5,231 (19.4)	2,729 (32.9)	<0.001
Medication for diabetes, n (%)	3 (1.4)	18 (2.7)	794 (1.4)	465 (1.1)	392 (1.5)	154 (1.9)	<0.001
Medication for dyslipidemia, n (%)	3 (1.4)	12 (1.8)	1,171 (2.0)	1,158 (2.8)	1,139 (4.2)	464 (5.6)	<0.001

Unless otherwise stated, the data presented are the means and standard deviations or the medians {first quartiles, third quartiles}. AST = aspartate transaminase; ALT = alanine aminotransferase; BMI = body mass index; eGFR = estimated glomerular filtration rate; FBG = fasting blood glucose; HDL-C = high-density lipoprotein cholesterol; LDL-C = low-density lipoprotein cholesterol; SBP = systolic blood pressure; SUA = serum uric acid; T-bil = total bilirubin; TC = total cholesterol; TG = triglyceride; TP = total protein.

**Table 2:** Comparison between subjects with hypouricemia and without hypouricemia.

**Men**			
	**SUA 0.0–2.0 mg/dL**	**SUA ≥ 2.1 mg/dL**	***P*** **Value**
	***n* = 235**	***n* = 110882**	
Age (years)	54.0 ± 14.1	51.6 ± 15.1	0.012
BMI (kg/m^2^)	23.1 ± 3.1	23.2 ± 3.1	0.420
SBP (mmHg)	125.1 ± 21.0	125.9 ± 19.7	0.526
TP (g/dL)	7.5 ± 0.5	7.5 ± 0.5	0.170
AST (IU/L)	24 {19,31}	24 {20,30}	0.802
ALT (IU/L)	18 {14,29}	19 {14,28}	0.323
T-bil (mg/dL)	0.83 ± 0.40	0.81 ± 0.36	0.347
TC (mg/dL)	193.3 ± 35.6	198.0 ± 35.7	0.042
TG (mg/dL)	98 {71,148}	103 {73,154}	0.220
LDL-C (mg/dL)	113.4 ± 30.7	116.6 ± 34.5	0.154
HDL-C (mg/dL)	56.0 ± 15.8	54.5 ± 14.7	0.101
FBG (mg/dL)	105.7 ± 32.4	102.0 ± 22.7	0.013
Current smoking, n (%)	102 (43.4)	49,826 (44.9)	0.637
Frequent alcohol drinker, n (%)	129 (54.9)	64,690 (58.3)	0.284
Medication for hypertension, n (%)	27 (11.5)	12,111 (10.9)	0.781
Medication for diabetes, n (%)	9 (3.8)	2337 (2.1)	0.067
Medication for dyslipidemia, n (%)	5 (2.1)	1462 (1.3)	0.278
eGFR (mL/min.1.73 m^2^)	83.4 ± 19.2	81.0 ± 17.0	0.029
**Women**			
	**SUA 0.0–2.0 mg/dL**	**SUA ≥ 2.1 mg/dL**	***P* value**
	***n* = 900**	***n* = 134906**	
Age (years)	50.2 ± 13.9	53.2 ± 14.5	<0.001
BMI (kg/m^2^)	21.8 ± 3.1	22.8 ± 3.3	<0.001
SBP (mmHg)	119.4 ± 20.7	123.0 ± 21.4	<0.001
TP (g/dL)	7.5 ± 0.5	7.6 ± 0.5	<0.001
AST (IU/L)	21 {18,25}	20 {16.25,24}	<0.001
ALT (IU/L)	13 {10,17}	14 {11,19}	<0.001
T-bil (mg/dL)	0.74 ± 0.32	0.74 ± 0.30	0.654
TC (mg/dL)	197.4 ± 35.0	206.2 ± 36.9	<0.001
TG (mg/dL)	74 {56.25,102}	84 {61,118}	<0.001
LDL-C (mg/dL)	118.6 ± 30.7	126.6 ± 33.4	<0.001
HDL-C (mg/dL)	61.8± 14.2	60.0 ± 14.6	<0.001
FBG (mg/dL)	99.1 ± 29.2	96.8 ± 18.0	<0.001
Current smoking, n (%)	46 (5.1)	6793 (5.0)	0.918
Frequent alcohol drinker, n (%)	66 (7.3)	11,588 (8.6)	0.180
Medication for hypertension, n (%)	96 (10.7)	17,624 (13.1)	0.033
Medication for diabetes, n (%)	21 (2.3)	1805 (1.3)	0.010
Medication for dyslipidemia, n (%)	15 (1.7)	3932 (2.9)	0.026
eGFR (mL/min.1.73 m^2^)	91.6 ± 20.3	81.6 ± 18.3	<0.001

Unless otherwise stated, the data presented are the means and standard deviations or the medians {first quartiles, third quartiles}. ALT = alanine aminotransferase; AST = aspartate transaminase; BMI = body mass index; eGFR = estimated glomerular filtration rate; FBG = fasting blood glucose; HDL-C = high-density lipoprotein cholesterol; LDL-C = low-density lipoprotein cholesterol; SBP = systolic blood pressure; SUA = serum uric acid; T-bil = total bilirubin; TC = total cholesterol; TG = triglyceride; TP = total protein.

**Table 3:** Comparison of lower hypouricemia group with the other groups in relation to the baseline characteristics.

**Men**						
	**SUA 0.0–1.3 mg/dL (A)**	**SUA 1.4–2.0 mg/dL (B)**	**SUA ≥ 2.1 mg/dL (C)**	**Comparison I (A) vs. (B)**	**Comparison II (A) vs. (C)**	**Comparison III (B) vs. (C)**
	***n* = 173**	***n* = 62**	***n* = 110,882**	***P* Value**	***P* Value**	***P* Value**
Age (years)	53.5 ± 13.7	55.6 ± 15.2	51.6 ± 15.1	0.295	0.099	0.033
BMI (kg/m^2^)	23.4 ± 3.1	22.0 ± 2.9	23.2 ± 3.1	0.002	0.365	0.002
SBP (mmHg)	124.9 ± 20.2	125.7 ± 23.1	125.9 ± 19.7	0.795	0.492	0.928
TP (g/dL)	7.5 ± 0.5	7.4 ± 0.4	7.5 ± 0.5	0.292	0.507	0.117
AST (IU/L)	25 {20,31}	23 {18,30.25}	24 {20,30}	0.337	0.452	0.442
ALT (IU/L)	19 {14,30.5}	18 {13.75,24.25}	19 {14,28}	0.344	0.751	0.162
T-bil (mg/dL)	0.82 ± 0.38	0.84 ± 0.45	0.81 ± 0.36	0.810	0.503	0.476
TC (mg/dL)	193.1 ± 34.5	193.6 ± 36.3	198.0 ± 35.7	0.936	0.073	0.327
TG (mg/dL)	104 {73.5,152.5}	88 {65.75,128}	103 {73,154}	0.045	0.935	0.024
LDL-C (mg/dL)	112.6 ± 29.9	115.5 ± 32.9	116.6 ± 34.5	0.523	0.130	0.807
HDL-C (mg/dL)	55.5 ± 15.8	57.5 ± 15.9	54.5 ± 14.7	0.413	0.339	0.109
FBG (mg/dL)	102.4 ± 26.9	114.9 ± 43.4	102.0 ± 22.7	0.009	0.802	<0.001
Current smoking, n (%)	77 (44.5)	25 (40.3)	49,826 (44.9)	0.568	0.910	0.465
Frequent alcohol drinker, n (%)	97 (56.1)	32 (51.6)	64,690 (58.3)	0.545	0.545	0.283
Medication for hypertension, n (%)	18 (10.4)	9 (14.5)	12,111 (10.9)	0.384	0.827	0.365
Medication for diabetes, n (%)	4 (2.3)	5 (8.1)	2,337 (2.1)	0.056	0.852	0.002
Medication for dyslipidemia, n (%)	5 (2.9)	0 (0.0)	1,462 (1.3)	0.329	0.070	0.363
eGFR (mL/min.1.73 m^2^)	81.9 ± 18.6	87.5 ± 20.6	81.0 ± 17.0	0.051	0.459	0.003
**Women**						
	**SUA 0.0–1.3 mg/dL (A)**	**SUA 1.4–2.0 mg/dL (B)**	**SUA ≥ 2.1 mg/dL (C)**	**Comparison I (A) vs. (B)**	**Comparison II (A) vs. (C)**	**Comparison III (B) vs. (C)**
	***n* = 221**	***n* = 679**	***n* = 134,906**	***P* Value**	***P* Value**	***P* Value**
Age (years)	52.1 ± 14.2	49.6 ± 13.7	53.2 ± 14.5	0.022	0.27	<0.001
BMI (kg/m^2^)	22.8 ± 3.7	21.4 ± 2.9	22.8 ± 3.3	<0.001	0.865	<0.001
SBP (mmHg)	121.7 ± 20.6	118.6 ± 20.7	123.0 ± 21.4	0.052	0.400	<0.001
TP (g/dL)	7.6 ± 0.5	7.5 ± 0.5	7.6 ± 0.5	0.026	0.600	<0.001
AST (IU/L)	21 {17,25}	19 {16,23}	21 {18,25}	<0.001	0.931	<0.001
ALT (IU/L)	15 {11,20}	12 {9,17}	14 {11,19}	<0.001	0.587	<0.001
T-bil (mg/dL)	0.75 ± 0.28	0.74 ± 0.33	0.74 ± 0.30	0.643	0.514	0.885
TC (mg/dL)	204.3 ± 35.2	195.2 ± 34.7	206.2 ± 36.9	<0.001	0.440	<0.001
TG (mg/dL)	84 {62.5,111}	70 {54,96}	104 {73,153}	<0.001	0.951	<0.001
LDL-C (mg/dL)	123.8 ± 33.2	116.9 ± 29.7	126.6 ± 33.4	0.004	0.218	<0.001
HDL-C (mg/dL)	61.1 ± 14.2	62.0 ± 14.2	60.0 ± 14.6	0.393	0.248	<0.001
FBG (mg/dL)	97.7 ± 18.6	99.5 ± 31.9	96.8 ± 18.0	0.429	0.449	<0.001
Current smoking, n (%)	12 (5.4)	34 (5.0)	6,793 (5.0)	0.804	0.789	0.974
Frequent alcohol drinker, n (%)	24 (10.9)	42 (6.2)	11,588 (8.6)	0.021	0.229	0.026
Medication for hypertension, n (%)	30 (13.6)	66 (9.7)	17,624 (13.1)	0.107	0.841	0.010
Medication for diabetes, n (%)	3 (1.4)	18 (2.7)	1,805 (1.3)	0.269	0.980	0.003
Medication for dyslipidemia, n (%)	3 (1.4)	12 (1.8)	3,932 (2.9)	0.679	0.169	0.076
eGFR (mL/min.1.73 m^2^)	83.4 ± 19.4	94.3 ± 19.9	81.6 ± 18.3	<0.001	0.139	<0.001

Unless otherwise stated, the data presented are the means and standard deviations or the medians {first quartiles, third quartiles}. ALT = alanine aminotransferase; AST = aspartate transaminase; BMI = body mass index; eGFR = estimated glomerular filtration rate; FBG = fasting blood glucose; HDL-C = high-density lipoprotein cholesterol; LDL-C = low-density lipoprotein cholesterol; SBP = systolic blood pressure; SUA = serum uric acid; T-bil = total bilirubin; TC = total cholesterol; TG = triglyceride; TP = total protein.

## Supplementary Materials

The following are available online at https://www.mdpi.com/1010-660X/55/3/61/s1, Table S1: Comparison of the baseline characteristics in men with serum uric acid levels of 0.0–1.3 mg/dL or 1.4–2.5 mg/d, Figure S1: Characteristics of each serum uric acid category in the context of cardiovascular risks according to sex.
