# Supplementary material for: Distribution and Characteristics of Hypouricemia within the Japanese General Population: A Cross-Sectional Study
Source: Medicina (Kaunas). 2019 Mar 4;55(3):61. doi: 10.3390/medicina55030061 (PMC6473785; doi:10.3390/medicina55030061)
Supplement: Supplementary file 1 [file medicina-55-00061-s001.pdf]

Table S1. Comparison of the Baseline Characteristics in the Men with Serum Uric Acid levels of 0.0–1.3 mg/dL or 1.4–2.5 mg/d

	SUA 0.0–1.3 mg/dL n=173	SUA 1.4–2.5 mg/dL n=296	*P* value
Age (years)	53.5 ± 13.7	56.7 ± 13.7	0.013
BMI (kg/m^2)	23.4 ± 3.1	21.8 ± 2.9	<0.001
SBP (mmHg)	124.9 ± 20.2	125.8 ± 20.6	0.652
TP (g/dL)	7.5 ± 0.5	7.4 ± 0.5	0.053
AST (IU/L)	25 [20, 31]	23 [19, 29]	0.089
ALT (IU/L)	19 [14, 30.5]	17 [13, 22]	0.019
T-bil (mg/dL)	0.82 ± 0.38	0.83 ± 0.41	0.854
TC (mg/dL)	193.1 ± 34.5	191.2 ± 35.6	0.553
TG (mg/dL)	104 [73.5, 152.5]	81.5 [62,117.75]	<0.001
LDL (mg/dL)	112.6 ± 29.9	112.7 ± 32.1	0.986
HDL (mg/dL)	55.5 ± 15.8	59.2 ± 15.7	0.017
FBG (mg/dL)	102.4 ± 26.9	113.6 ± 45.2	0.003
Current smoking, n (%)	77 (44.5)	124 (41.9)	0.581
Frequent alcohol drinker, n (%)	97 (56.1)	148 (50.0)	0.204
Medication for hypertension, n (%)	18 (10.4)	39 (13.2)	0.376
Medication for diabetes, n (%)	4 (2.3)	16 (5.4)	0.154
Medication for dyslipidemia, n (%)	5 (2.9)	3 (1.0)	0.152
eGFR (mL/min.1.73m^2)	81.9 ± 18.6	88.9 ± 20.9	0.003

Unless otherwise stated, the data presented are the means and standard deviations or the medians (first quartiles, third quartiles).

ALT = alanine aminotransferase; AST = aspartate transaminase; BMI = body mass index; eGFR = estimated glomerular filtration rate; FBG = fasting blood glucose; HDL-C = high-density lipoprotein cholesterol; LDL-C = low-density lipoprotein cholesterol; SBP = systolic blood pressure; SUA = serum uric acid; T-bil = total bilirubin; TC = total cholesterol; TG = triglyceride; TP = total protein.
